# Fast and Sensitive Analysis of Cefiderocol in Human Plasma Microsamples by Liquid Chromatography-Isotope Dilution Tandem Mass Spectrometry for Therapeutic Drug Monitoring

**DOI:** 10.3390/antibiotics12020213

**Published:** 2023-01-19

**Authors:** Rossella Barone, Matteo Conti, Pier Giorgio Cojutti, Milo Gatti, Pierluigi Viale, Federico Pea

**Affiliations:** 1 Clinical Pharmacology Unit, IRCCS Azienda Ospedaliero Universitaria of Bologna, 40138 Bologna, Italy; 2 Department of Medical and Surgical Sciences, Alma Mater Studiorum, University of Bologna, 40138 Bologna, Italy; 3 Infectious Diseases Unit, IRCCS Azienda Ospedaliero Universitaria of Bologna, 40138 Bologna, Italy

**Keywords:** cefiderocol, therapeutic drug monitoring, plasma microsamples, Liquid Chromatography-Isotope Dilution Tandem Mass Spectrometry

## Abstract

Cefiderocol (C) is a parenteral siderophore cephalosporin with relevant inter-individual pharmacokinetic variability among critically ill patients, which may potentially affect effective drug exposure. Therapeutic drug monitoring (TDM) may concur in improving the real-time management of C therapy in clinics. In this study, we developed and validated a fast and sensitive Liquid Chromatography-Isotope Dilution Tandem Mass Spectrometry (LC-ITD-MS/MS) method for measuring C in human plasma microsamples, as small as 3 microliters. Analysis was preceded by a user-friendly pre-analytical single-step and was performed by means of a very fast chromatographic run of 4 min, followed by positive electrospray ionization and detection on a high sensitivity triple quadrupole tandem mass spectrometer operated in multiple reaction monitoring mode. The straightforward analytical procedure was successfully validated, based on the European Medicines Agency (EMA) guidelines, in terms of specificity, sensitivity, linearity, precision, accuracy, matrix effect, extraction recovery, limit of quantification, and stability. The novel method was successfully applied to TDM of C in more than 50 cases of critically carbapenem-resistant Gram-negative bacterial infections and enabled us to optimize antibiotic therapy.

## 1. Introduction

**Figure 1:**
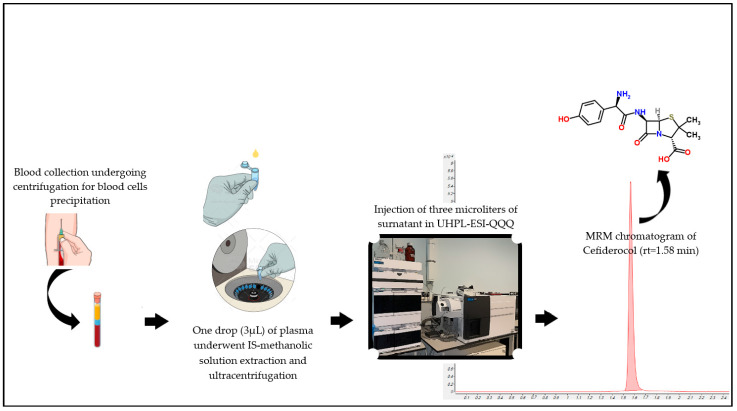
Cartoon depicting the analytical workflow employed in the analysis of Cefiderocol (chemical structure on the upper right corner) starting from a small plasma droplet obtained by centrifugation of whole blood.

Infections caused by Carbapenem-Resistant *Enterobacterales* (CRE) and non-fermentative Gram-negative pathogens (such as *Pseudomonas aeruginosa*, *Acinetobacter baumannii*, and *Stenotrophomonas maltophilia*) are currently a major global health concern, accounting for remarkable hospital morbidity and mortality [1]. Among the different novel beta-lactams recently licensed for the management of Difficult-To-Treat (DTR) Gram-negative infections [2], cefiderocol (C, chemical structure in Figure 1) represents a promising option, considering its valuable in vitro activity against CRE, DTR *Pseudomonas aeruginosa*, carbapenem-resistant *Acinetobacter baumannii*, and *Stenotrophomonas maltophilia* [3,4].

However, the efficacy of cefiderocol in the management of carbapenem-resistant *Acinetobacter baumannii* infections has been questioned by the CREDIBLE-CR trial that showed that both clinical and mortality rates were higher in patients treated with cefiderocol compared to those treated with best available therapy [5], and both ESCMID guidelines [6] and IDSA guidance [7] recommended against the use of this agent in carbapenem-resistant *Acinetobacter baumannii* scenarios. Conversely, some real-world clinical data suggested that cefiderocol may have good efficacy in the treatment of infections caused by this pathogen [8,9]. Notably, in a case series of 13 critically ill patients treated with cefiderocol for documented ventilator-associated pneumonia and/or bloodstream infections due to carbapenem-resistant Acinetobacter baumannii, a trend toward a proportional increase in microbiological eradication rate was found when the pharmacokinetic/pharmacodynamic target attainment shifted from suboptimal to quasi-optimal and optimal [10], thus suggesting the potential remarkable role of real-time therapeutic drug monitoring of cefiderocol in challenging clinical scenarios. Currently, some analytical methods for measuring C were developed by means of High Performance Liquid Chromatography (HPLC) coupled to UltraViolet (UV) [11,12] or to electrochemical detection [13]. HPLC coupled to tandem mass spectrometry (LC-MS/MS) methods has been developed for C alone [14] and for the simultaneous determination of C and Ceftobiprole [15]. All of these methods showed good performance and may enable C determination in plasma samples of at least 50 microliters or more.

The use of plasma microsampling techniques may have some practical advantages compared to conventional venipuncture methods [16]. Microsampling enables reducing stress and pain related to venipuncture procedures and may be particularly advisable in fragile populations [17,18]. Blood micro-samples may be collected onto specialty paper(s) [19], polymeric tips, or capillaries [16] and may even be directly collected onto dried plasma spots (DPS) [20]. Blood droplets can also be collected in small dedicated vials, and plasma can be obtained by centrifugation in the laboratory.

The aim of this study was to develop and validate a fast, selective, and simple method for measuring C in human plasma microsamples of 3 microliters by means of LC-MS/MS, supporting real-time therapeutic drug monitoring.

## 2. Results

### 2.1. Optimization of LC-MS/MS Conditions

Single charge positive ion mass transitions for optimal sensitivity and specificity were selected at 752.1–285.2 and 764.1–297.3 for C and [2H12]-C as Internal Standard (IS), respectively, by scrutinizing the MS/MS fragmentation pattern spectra of analytes and by comparing with those reported in the literature [15,21]. Singly charged molecular ions (M+H+) showed higher (approximately double) signal intensity compared to the doubly charged adduct (M+2H2+). The optimization of Multiple Reaction Multiple (MRM) signals gave the results reported in Table 1.

The LC optimization started with a standard gradient elution, as described in Table 2. The elution program through the ZORBAX Eclipse plus C18 column was based on simple mobile phases consisting of (A) water-formic acid (100:0.1, *v*/*v*) and (B) methanol-formic acid (100:0.1, *v*/*v*). It enabled to achieve good quality and shape of the chromatographic peak in a chromatographic run time as short as 4 min without affecting peak performance. The retention time (rt) of 1.58 min was very reproducible, thus confirming optimal column reconditioning throughout runs. A 0.5-min reconditioning step at 0.5 mL/min flow of 5% B mobile phase (see Table 1) was long enough for proper column reconditioning between runs.

**Figure 2:**
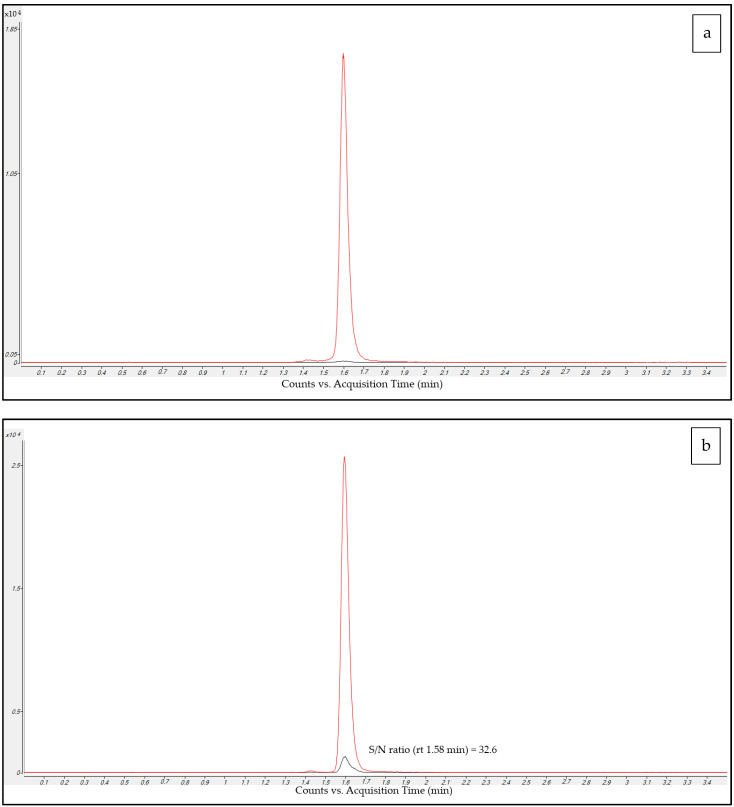

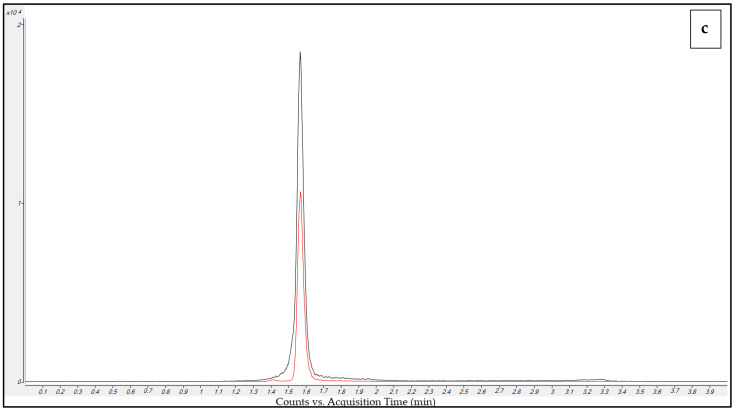
Overlayed MRM chromatograms for C (black) and C-d12 (red) obtained in the analysis of (**a**) a blank sample extracted with the methanol-IS solution, showing the absence of peaks related to C and the presence of a well-defined peak for C-d12; (**b**) a LOQ sample with printed S/N ratio (SNR); (**c**) a real patient sample showing good peak shape and resolution of specific peaks both for C standard and C-d12.

Drug-free plasma sample MRM chromatograms (Figure 2a) obtained after injecting IS-methanol solution extracts showed the presence of only [2H12]-C peak whereas C peak were always below the detection limit. This allows us to confirm MRM transitions’ specificity and the purity of [2H12]-C standard solution.

The Limit of Quantification (LOQ) sample MRM chromatograms (Figure 2b) showed a value of 32.6 for the signal to noise (S/N) ratio of the C peak, and this may support the high sensitivity of the method. The LOQ of the method could be much lower than the one that in current validation we set equal to the lower calibrator (0.1 mg/L).

Real sample MRM chromatograms (like the example in Figure 2c) showed sharp peak shape and excellent resolution, even at low concentrations. No isobaric peaks were observed in samples, confirming the selectivity of the employed MRM transitions. This section may be divided by subheadings. It should provide a concise and precise description of the experimental results, their interpretation, and the experimental conclusions that can be drawn.

### 2.2. Method Validation

#### 2.2.1. Sensitivity

The LOQ was 0.1 mg/L and showed a favorable signal to noise (S/N) = 32.6 and corresponded to the lowest point in the calibration curve and (Figure 2b).

#### 2.2.2. Selectivity and Carry-Over

MRM chromatograms of C and [2H12]-C in the ten plasma pool samples collected from hospitalized patients under various therapies showed no interfering peaks at the retention time of the analyte nor at any other retention time (example in Figure 2c), supporting the high degree of specificity of the LC-MS/MS method. MRM chromatograms of C-free plasma samples run after the Upper Limit of Quantification (ULOQ) sample showed peak areas below 20% of that of the LOQ, attesting to a lack of significant carry-over between runs, most probably thanks to the very low injection volume (3 μL) used in this method.

#### 2.2.3. Linearity and Limit of Quantification (LOQ)

**Figure 3:**
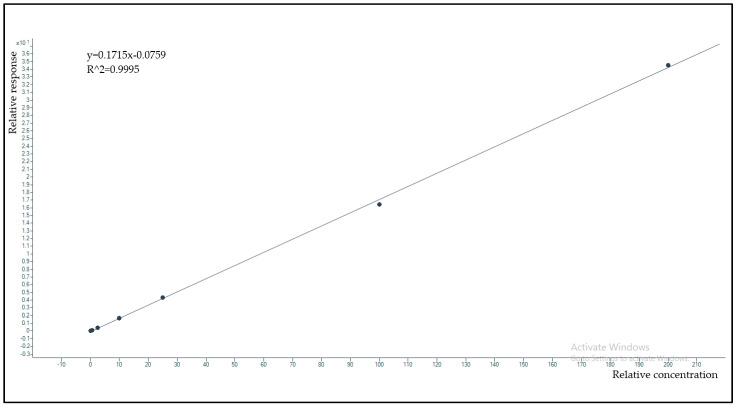
Example of a calibration obtained by plotting the C/C-d12 area ratio (response) over concentration, in the 0.1200.0 mg/L range, by software fitting of the 7 experimental calibration points with the linear equation and correlation coefficient reported in the upper left corner of the box, both indicating stringent linearity of the calibration model.

The calibration curve model using response (C peak area / [2H12]-C peak area) over concentration showed good data fitting (Figure 3). The equation calculated by pooled data coming from seven different days was y = 0.172 (±0.006) × −0.081 (±0.003). The average regression coefficient was R^2^ = 0.997 (±0.002), confirming strict linearity of the calibration curve. Since the clinical sample concentrations never exceeded the ULOQ of 200 mg/L, the dilution integrity with a drug-free matrix was not tested for this method.

#### 2.2.4. Accuracy and Precision

The precision (mean CV %) and accuracy (mean BIAS%) results are shown in Table 3; all met the EMA requirements. The intra- and inter-day coefficients of variation of the different Quality Control (QC) levels ranged from 7.5% to 13.5% and from 8.0% to 14.8%, respectively. The intra- and inter-day accuracy bias of Low Quality Control (LQC), Medium Quality Control (MQC), and High Quality Control (HQC) concentration levels ranged from 7.9% to 9.3% and from 8.3% to 13.1%, respectively.

#### 2.2.5. Matrix Effect and Extraction Recovery

The Percent Matrix effect (%ME) and Percent Extraction Recovery yield (%ER) were calculated at Low, Medium, and High concentration levels (Table 4). A signal enhancement effect was observed at all of the tested concentrations, and this further increased the method sensitivity. When normalization by the internal standard area was applied, the matrix effect values matched the criteria fixed by the EMA for validation.

The extraction recovery yield ranged from 76.3 to from 86.6 and satisfied EMA criteria. However, its wide variability observed under the different tested concentrations, albeit below 10.3 %, pointed out the need to add an IS for accurate quantification throughout the whole dynamic range.

#### 2.2.6. Stability

C stability was tested at all the QC levels in different operating conditions, as specified in Table 5. After just one freeze thawing cycle, a decrease in C concentration was observed at all of the tested levels, pointing out a limitation in the possibility of sample reprocessing. The autosampler extracts were stable for less than 2 h, despite the fact that the autosampler was kept at 10° C, posing quite strict limits in the possibility of reanalyzing sample extracts. Autosampler extracts were stable if kept frozen at −20° C for 24 h.

### 2.3. Clinical Application

**Figure 4:**
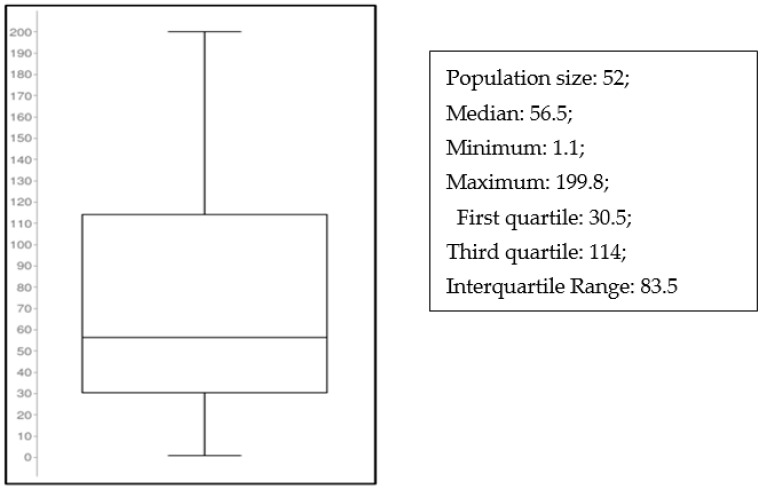
Box plot showing the spread of the C concentration measured in 52 real patients treated because of carbapenem-resistant Gram-negative infections.

C concentrations were measured in blood samples coming from 52 patients who were treated with C because of carbapenem-resistant Gram-negative infections. All of these were within the calibration range that was selected for the ITD LC-MS/MS method here described. In our study, C treatment was administered according to its technical protocol (considering patients’ conditions, such as renal disfunction). The distribution of C plasma concentrations observed in more than 50 critically ill patients who underwent real-time TDM is showed in the Figure 4 (Boxplot).

## 3. Discussion

An accurate, precise, and sensitive bioanalytical method for measuring C in plasma microsamples of 3 microliters collected from patients under therapy was developed.

The sample preparation was straightforward, and a simple procedure of plasma dilution with water (1:17 *v*/*v*) and protein-crash with methanol (1:3 *v*/*v*) was sufficient to obtain clean sample extracts with a good extraction yield (of about 80%) when analyzed by LC-MS/MS. Other authors utilized different sample preparations for the LC-MS/MS analysis, such as: (1) plasma protein precipitation with a 30% sulphosalicylic acid solution [15], (2) ultra-filtration to isolate plasma water containing unbound C, which is the prevalent fraction [14]. Our preparation method simultaneously extracts and precipitates proteins and therefore cannot discriminate between C free or bound fraction. This limitation of our preparation method, however, was not meaningful in our practice since the total plasma concentration was considered for TDM optimization. Moreover, the total plasma concentrations observed by us (see Figure 4) were in the same range as those observed in the literature with other methods, confirming that the bound fraction is indeed a minor component of C in plasma.

Method selectivity is mainly influenced by selected MRM transitions in LC-MS/MS methods. We therefore employed the same MRM transition used by Llopis et al. [15], with noteworthy performance. In fact, absolute selectivity was confirmed by the absence of interfering peaks in MRM chromatograms relative to 10 plasma pool samples obtained by mixing aliquots from hundreds of patients under various drug therapies with the exception of C.

The analytical sensitivity was equal to those of another published LC-MS/MS method [15] but was obtained in much smaller samples (3 vs. 100 microliters), indicating a better absolute sensitivity. The LOQ adopted for validation purpose (0.1 mg/L) is well in line with the need for efficient TDM [11]. The ion enhancement matrix effect of about 180 % (Table 4) favors high analytical sensitivity.

It is noteworthy that chromatographic run time is very fast (4 min), like that of other LC-MS/MS methods [15,22], and is much faster than those reported previously for HPLC-UV methods (12–15 min) [11].

The precision and accuracy of the method are demonstrated by low inter- and intra-day %C vs and bias% (Table 3), which are mandatory for efficient clinical TDM evaluations. In addition, the linearity over the whole dynamic range from 0.1–200 mg/L, lower than Llopis (1–200 mg/L) [15], allows for the direct processing of all clinically meaningful samples without the need to reanalyze highly concentrated samples after dilution. This is an interesting feature of the method, which can contribute to pushing laboratory productivity when considering the wide inter patient PK variability observed under C treatment (Figure 4).

One major limit to keep in mind in C analytical determinations is the short stability of C aqueous solutions [23], a well-known issue in the literature that was confirmed in our experimental stability tests (Table 5). In fact, this kind of instability poses constraints in laboratory operations when performing C analysis. To deal properly with this issue, healthcare workers in our hospital have been instructed that blood samples must be dispatched to the lab immediately after collection and then processed without any delay or, alternatively, frozen immediately at −80 °C. Plasma samples are stable for at least 2 months at −80 °C, if any thawing occurred (data not shown). However, C deteriorates rapidly even after just one freeze thawing cycle, so an accurate assessment of C concentrations in plasma samples may be performed only once. A limitation of the presented method is therefore the lack of a procedure to reduce the physico-chemical degradation of C during analysis. The addition of a stabilizer molecule in vials just after blood withdrawal, for instance, could greatly improve reliability of analytical determinations. Experiments are ongoing to find suitable stabilizers.

The widespread distribution of C plasma concentrations that were observed in more than 50 critically ill patients who underwent real-time TDM may support the need for individualizing C exposure in each single patient [10,11,21,24].

## 4. Materials and Methods

### 4.1. Chemical and Reagents

C and deuterated cefiderocol ([2H12]-C) powders were provided by Shionogi & Co, Ltd. (Osaka, Japan) (Figure 1). All reagents were purchased from CHROMASOLV™ (Thermofisher Scientific, Milan, Italy) and had the highest available analytical grades. Liquid chromatography–MS/MS grade water (ultrapure water) was produced by a Milli-Q^®^ Direct system (Millipore Merck—Darmstadt, Germania) and drug-free plasma from healthy donors was supplied by the IRCCS Azienda Ospedaliero Universitaria di Bologna (Bologna, Italy).

### 4.2. Stock Solutions, Standards and Quality Controls

C’s stock solution was prepared in MilliQ water at a concentration of 100 mg/mL. Calibrators were obtained by spiking drug-free plasma from previously nominated stock, stored at room temperature for at least 2 h for enabling equilibration process prior to use. Another stock solution in ultrapure water was used for preparing independent quality control (QC) samples.

The calibration curve ranged from 0.1 to 200 mg/L (calibration points: 0.1–0.5–10–25-100–200 mg/L). Four QC samples were set at 0.1 mg/L (Lower Limit of Quantification, LOQ), 0.25 mg/L (Low QC, LQC), 75 mg/L (Medium QC, MQC), and 150 mg/L (High QC, HQC). A solution of 0.5 mg/L ([2H12]-C) in methanol was used as internal standard. All solvents and matrix solutions were stored at −80 °C.

### 4.3. Instrumentation

Chromatography was performed by means of an Agilent 1295 U-HPLC coupled with an autosampler kept at 10 °C and with a ZORBAX Eclipse plus C18 column (2.1 × 50 mm, 1.8 µm particle size; (Agilent, Santa Clara, CA, USA)) kept at 25 °C. Analyte separation was obtained over 4 min by means of a binary pump program with linear flow gradient elution from mobile phases A [water-formic acid (100:0.1, *v*/*v*)] to mobile phase B [methanol -formic acid (100:0.1, *v*/*v*)] at a flow rate of 0.5 mL/min, as described in Table 1. The UHPLC system was coupled with a triple quadrupole mass spectrometer: 6495c (Agilent, Santa Clara, CA, USA). Acquisitions were achieved in Multiple Reactions Monitoring (MRM) mode, and electrospray ionization (ESI) was operated in positive mode. MS/MS parameters were set as follows: gas temp = 200 °C, gas flow = 14 L/min, nebulizer pressure = 35 psi, sheath gas temp = 300 °C, sheath gas flow = 11 l/min, capillary voltage = 4000 V, nozzle voltage = 0 V. MRM parameters (Table 2). Chromatographic data acquisition, peak integration and quantification were performed by means of the MassHunter software Agilent, Santa Clara, CA, USA).

### 4.4. Sample Pre-Treatment

Sample preparation was performed by adding 47 µL of ultrapure water to 3 µL of human plasma and then mixed with 150 μL of the IS-methanol solution. The mixture was vortexed for 15 s and centrifuged for 5 min at 13,000 rpm at room temperature. Subsequently, 100 µL of the clear supernatant was transferred to an autosampler vial, and 3 µL was injected into the LC-MS/MS system.

### 4.5. Method Validation

Method was validated in agreement with the European Medicines Agency (EMA) guidelines for developing bioanalytical methods [25]. Selectivity, linearity, accuracy, precision, limit of quantification (LOQ), recovery, matrix effect, and stability were evaluated [25].

#### 4.5.1. Selectivity and Carry-Over

Ten different plasma samples were analyzed to test the lack of response at the retention times of C and [2H12]-C from endogenous components in the matrix or other components. The carry-over effect was evaluated by injecting blank plasma samples after calibration standard at the upper limit of quantification, and considered negligible if the signal was lower than 20% of the method’s LOQ.

#### 4.5.2. Linearity and Limit of Quantification (LOQ)

Six plasma calibrators were created by spiking blank matrices with C and [2H12]-C over the range from 0.1 to 200 mg/L. Linearity was defined if linear regression coefficient (R) 2 was ≥0.9985. The LOQ was the lower concentration covered by the dynamic range which showed a signal to noise ratio (S/N) higher than 10.

#### 4.5.3. Precision and Accuracy

Precision (mean CV%) and accuracy (mean BIAS%) were calculated by analyzing LOQ, LQC, MQC and HQC five times (intra-day) in three different inter-day analytical runs.

#### 4.5.4. Matrix Effect and Extraction Recovery

Percent Matrix effect (ME) and Extraction Recovery (ER) were estimated at three QC levels (Low, Medium, and High) by means of the following equations:

ME (%) = B/A × 100(1)

ER (%) = C/B × 100(2)

where:

A = C/[2H12]-C peak areas obtained by injecting water-methanol 1:3 *v*/*v* samples (*n* = 3) spiked at the three concentration levels.

B = C/[2H12]-C peak areas obtained by a drug-free plasma extract (*n* = 3) spiked at the three concentration levels after the extraction.

C = C/[2H12]-C peak areas obtained by drug-free plasma (*n* = 3) spiked at the three concentration levels before extraction.

This was performed on ten different patients’ plasma samples to properly manage individual matrix composition variability.

#### 4.5.5. Stability

The stability of C in human plasma and its extract was performed in different storage conditions and at different concentrations in the calibration range (low, medium and high QC levels). Based on specific laboratory needs and routine, we tested:

- extracts on board kept at 10 °C during 24 h;
- extracts kept at −20 °C during 24 h;
- matrix samples after three complete freeze and thaw cycles from −80 °C to 25 °C.

For testing, sample concentrations before and after storage were compared with the nominal concentrations. C was considered to be stable in plasma samples and extracts under different storage conditions if measured concentrations were within ±15% of the nominal concentrations.

### 4.6. Clinical Application

The presented ITD LC-MS/MS method was tested for measuring C concentrations in 52 patients treated because of carbapenem-resistant Gram-negative infections, as previously reported [26,27]. Plasma samples were processed immediately after delivery or after freezing at −80 °C until analysis, depending on a case by case situation.

## 5. Conclusions

In conclusion, a fast, sensitive, accurate LC-MS/MS method for quantifying C in human plasma microsamples was developed and validated. Thanks to its high performance and rapid execution, this method may be favorably used for real-time TDM purposes in the clinic environment.

## Figures and Tables

**Table 1:** Specific MRM transition parameters used for Cefiderocol and Cefiderocol-d12 (IS) acquisition.

Analyte	Retention Time (min)	Precursor Ion (*m/z*)	Product Ion (*m/z*)	Dwell Time (ms)	Fragmentator (V)	Collision Energy (V)
Cefiderocol	1.58	752.1	285.2	50	166	20
Cefiderocol-d12	1.58	764.1	297.3	50	166	20

**Table 2:** Binary pump program used for linear gradient elution with mobile phases A and B.

Time (min)	A (%)	B (%)	Flow (mL/min)
0	95	5	0.5
2	5	95	0.5
2.5	5	95	0.5
2.51	95	5	0.5
4	95	5	0.5

**Table 3:** Intra-day and inter-day average (avg) precision and accuracy assessed at four concentration levels (LOQ, LQC, MQC, and HQC) five times (intra-day) in three different analytical runs (inter-day).

QC Levels		Intraday (*n* = 5)			Inter-Day (*n* = 3)		
Sample Name	Nominal Conc. (mg/L)	Avg Conc. (mg/L)	Avg Accuracy (Bias%)	Avg Conc. (mg/L)	Avg Conc. (mg/L)	Avg Precision (CV%)	Avg Accuracy (Bias%)
LOQ	0.1	0.12	13.5	8.8	0.12	14.8	9.2
LQC	0.25	7.5	10.9	9.3	9.8	9.6	8.3
MQC	75	102.5	9.8	7.9	104.2	8.0	9.7
HQC	150	395.7	9.7	8.1	401.1	11.0	13.1

**Table 4:** Average (Avg) Matrix effect (ME%) and Recovery (ER%) of C measured at different concentration levels.

Quality Control Level	N° Replicates	Average Matrix Effect (%)	Average IS-Normalized Matrix Effect (%)
LQC	30	181.9	104.2
MQC	30	185.7	105.1
HQC	30	187.2	98.3

**Table 5:** Stability of C at different storage conditions. In our study, we tested both the extracts and the plasma samples (according to our routine needs).

Quality Control		LQC	MQC	HQC
Types of sample	Tested conditions	Avg Accuracy (Bias%)	Avg Accuracy (Bias%)	Avg Accuracy (Bias%)
extracts	autosampler post 2 h	−22.1	−29.1	−24.6
	freezer post 24 h	−16.5	−16.7	−22.1
plasma samples	freeze-thaw stability			
	1 cycle	−15.8	−15.1	−15.4
	2 cycle	−35.1	−39.0	−32.6
	3 cycle	−77.4	−75.2	−76.4

## Data Availability

The data presented in this study are available on request from the corresponding author. The data are not publicly available due to privacy concerns.
